# Interleukin-13 Treatment of Living Lung Tissue Model Alters the Metabolome and Proteome—A Nano-DESI MS Metabolomics and Shotgun Proteomics Study

**DOI:** 10.3390/ijms25095034

**Published:** 2024-05-05

**Authors:** Gábor Tóth, Anastasia Golubova, Alexander Falk, Sara Bergström Lind, Mark Nicholas, Ingela Lanekoff

**Affiliations:** 1 Department of Chemistry—BMC, Uppsala University, 75237 Uppsala, Sweden; 2 AstraZeneca R&D, 43183 Mölndal, Sweden; carlisleohio@gmail.com

**Keywords:** asthma, interleukin-13, nano-DESI, proteomics, metabolomics, multi-omics, air–liquid interface, amino acid

## Abstract

Asthma is a chronic respiratory disease with one of the largest numbers of cases in the world; thus, constant investigation and technical development are needed to unravel the underlying biochemical mechanisms. In this study, we aimed to develop a nano-DESI MS method for the in vivo characterization of the cellular metabolome. Using air–liquid interface (ALI) cell layers, we studied the role of Interleukin-13 (IL-13) on differentiated lung epithelial cells acting as a lung tissue model. We demonstrate the feasibility of nano-DESI MS for the in vivo monitoring of basal–apical molecular transport, and the subsequent endogenous metabolic response, for the first time. Conserving the integrity of the ALI lung-cell layer enabled us to perform temporally resolved metabolomic characterization followed by “bottom-up” proteomics on the same population of cells. Metabolic remodeling was observed upon histamine and corticosteroid treatment of the IL-13-exposed lung cell monolayers, in correlation with alterations in the proteomic profile. This proof of principle study demonstrates the utility of in vivo nano-DESI MS for characterizing ALI tissue layers, and the new markers identified in our study provide a good starting point for future, larger-scale studies.

## 1. Introduction

Asthma is a chronic respiratory disease, affecting millions of people around the world [1]. It causes inflammation, bronchial hyperresponsiveness, and remodeling of the airways, which result in shortness of breath, wheezing, and coughing [2]. The pathophysiology of asthma is reported to be related to the activity of T-helper 2 (Th2) lymphocytes. Particularly in allergic asthma, Th2 lymphocytes produce Th2-derived cytokines (interleukins, IL) IL-4, IL-5, IL-9, and IL-13 that promote the development of inflammatory cells, including mast cells [3]. Out of these cytokines, IL-13 is a central mediator of allergic asthma and therefore one of the most important cytokines for asthma development and pathogenesis [4]. For example, IL-13 can induce airway remodeling, the production of inducible nitric oxide synthase (iNOS), mucus secretion, and the release of inflammatory mediators from mast cells, including histamine [5,6]. Histamine is the first chemical mediator found to evoke bronchial smooth muscle contraction in asthma, and it plays a key role in asthma development by triggering proinflammatory responses in tissue and eliciting major responses involved in allergic reactions [7]. High histamine levels have been found to correlate with asthma severity [5], which makes it an interesting target to study. A major treatment option for asthmatic attacks is the use of inhaled corticosteroids, such as fluticasone, budesonide, and terbutaline. Although they are found to increase lung function and reduce inflammation during long-term use in most patients [8,9,10], they are not ultimately effective for all patients. Therefore, therapies that target interleukins with monoclonal antibodies have emerged during the last decade, albeit with varying efficacy on lung function [11]. Thus, new opportunities for the development of treatments are required.

To gain a clear understanding of the alterations related to asthma, it is critical to obtain a deeper knowledge of the cellular and molecular mechanisms that correlate with the disease. Airway epithelia are commonly studied using a living lung tissue model of primary human bronchial epithelial cells (PBECs) cultured in an air–liquid interface (ALI) [12,13,14]. In the ALI system, the PBECs are placed in a culture medium on a semipermeable surface. After some time, the growth medium on the apical side of the cells is removed, creating the ALI system. In the ALI system, the cells differentiate into a pseudostratified cell layer, consisting of ciliated cells, goblet/secretory cells, and basal cells [15]. This mimics the in vivo conditions of the lung, with the apical side of the ALI system towards the air and the basolateral side on the semipermeable membrane in contact with the culture medium [15,16].

The ALI lung tissue model can be transformed into an asthma model by introducing IL-13 to the medium on the basolateral side [17]. Multiple studies using the ALI model have reported that IL-13 stimulation alters the cell differentiation process, generates more mucus cells and fewer ciliated cells, and increases paracellular permeability [18,19]. Additionally, gene expression studies have found that IL-13 induces the hypoxia signaling pathway [20] and the eicosanoid metabolism that leads to a reduction in airway prostaglandin E2 [21]. Furthermore, the addition of histamine to the media is considered to imitate an allergic asthma attack, where the inhaling of allergens causes intensive histamine production and then bronchial contraction and intensive mucus secretion [5]. Overall, it is clear that IL-13-treated ALI-cultured cells constitute a living lung model to study asthma-relevant effects.

Metabolomics is a powerful tool that can reveal altered metabolic pathways due to stress conditions such as pathogenesis or drug treatment [22,23,24]. Previous studies have investigated metabolome alterations in ALI cells [25,26,27]. In such studies, the tissue model is exposed to drugs or smoke for one or several time periods. The metabolic responses are then studied either by first lysing the cells or by covering the apical side with a medium that is then removed for analysis. However, covering the apical side with medium decreases oxidative metabolism on both the apical and the basolateral side [28]. Furthermore, cell lysis for each time point increases the number of culture inserts. Thus, there is a need for a method that enables time-resolved metabolomics of ALI-cultured cells in a non-destructive manner. The ability to directly analyze the apical side of the living cellular layer would permit the study of molecular transport and the metabolic responses that occur upon IL-13 remodeling. Using the ALI interface also allows for subsequent proteomics characterization which opens the way to multi-omics characterization of ALI cell layers. This is especially important, as “bottom-up” proteomics has become one of the main techniques of biochemical profiling and drug discovery in the past decade [29]. In vivo studies of the cell cultures without sample preparation contribute to understanding molecular alterations, providing valuable information about cellular activity and physiology, and facilitating biomarker research for clinical diagnostics [30].

The main aim of this study was to perform an in situ and temporal analysis of both the endogenous and basally-introduced molecules that are transported and metabolized by the living lung tissue model. This was achieved using nanospray desorption electrospray ionization mass spectrometry (nano-DESI MS), where the probe of two fused silica capillaries enables localized liquid extraction and subsequent nanoESI MS directly from the living cell layer. Here, we present time-resolved nano-DESI MS metabolite profiling of living ALI lung epithelial cells under native and IL-13-induced conditions and upon treatment with histamine and corticosteroids. Subsequently, we analyze the proteome and use the multi-omics results to characterize the chemical alterations upon IL-13 treatment, linking to asthma pathophysiology.

## 2. Results

### 2.1. Air–Liquid Interface Nano-DESI MS for Direct Analysis of Living Cells

**Figure 1:**
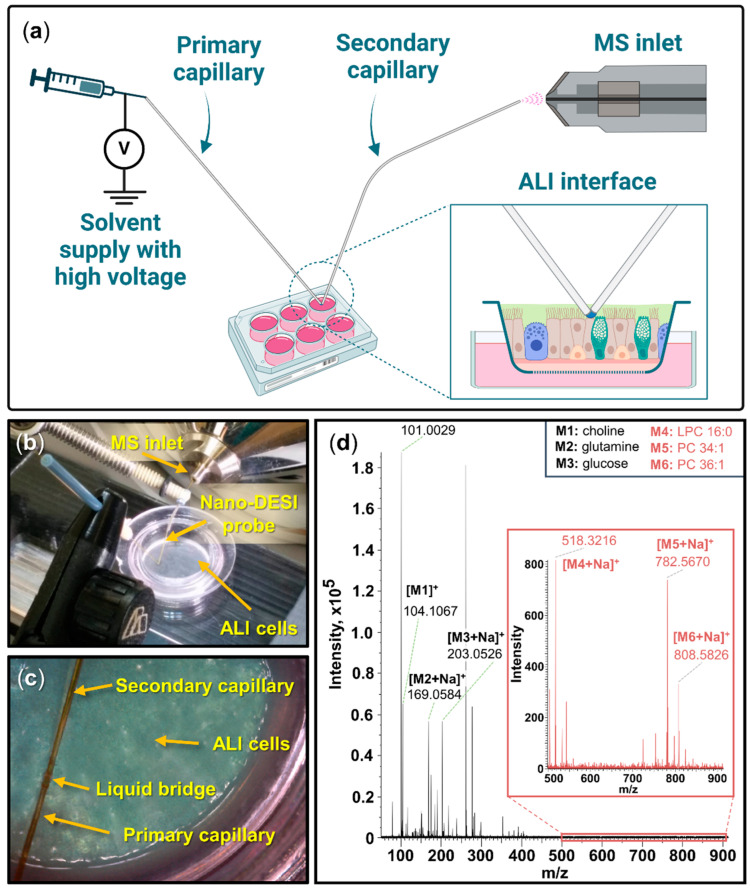
Nano-DESI MS setup for sampling the metabolome of living ALI cell surface. (**a**) Schematic of the experimental setup for sampling of the cellular layer in the microwells with nano-DESI MS. The illustration is not to scale. (**b**) Side-view picture of sampling the ALI interface with nano-DESI MS. (**c**) Zoomed-in view of sampling the ALI interface. (**d**) Averaged mass spectrum acquired from the mucus of the living cells showing a wide range of detected endogenous metabolites and low signal of phospholipids (insert). Panel (**a**) was created with Biorender.com.

An air–liquid interface (ALI) model was used to imitate the epithelium cell layer in the lungs; the cells were placed between an air environment and a liquid medium on a porous filter. Thereafter, nano-DESI was used for the direct sampling of molecules from living cells. For sampling the endogenous metabolome, the nano-DESI probe was placed directly in contact with the ALI cells, and the molecules were desorbed into the liquid bridge between the two capillaries and subsequently electrosprayed into the MS (Figure 1a–c).

In contrast to the conventional nano-DESI setup, the secondary capillary was bent about 45 degrees to allow for reaching the cellular layer in the ALI wells. The typical spot size of the nano-DESI probe is 50–100 µm [31,32] and the diameter of one epithelium cell is up to 20 µm [33], suggesting that analyses represent an average from a group of cells. We performed short-time (<1 min) “touch-down” experiments on restricted sampling areas. The short time and the high surface sensitivity of the sampling probe ensured that cell damage caused by the high methanol content was avoided. The short time caused the sampling to mostly be of the mucus and not the cells. In test experiments of longer sampling times, the amount of phosphatidylcholine species extracted from the cell membranes increased significantly. Thereby, by the short sampling, we ensured that the cellular layer was kept intact without leakage of the medium through the cellular layer. Furthermore, each sampling position was a different spot on the cellular layer, limiting possible bias in the study due to induced stress conditions. In a typical mass spectrum acquired from the living cellular surface, metabolites such as choline [M]^+^ (*m/z* 104.1067), glutamine [M+Na]^+^ (*m/z* 169.0584), and glucose [M+Na]^+^ (*m/z* 203.0526) were detected (Figure 1d). The minute to non-detectable signal of phospholipids, such as LPC 16:0 [M+Na]^+^ (*m/z* 518.3216), PC 34:1 [M+Na]^+^ (*m/z* 782.5670), and PC 36:1 [M+Na]^+^ (*m/z* 808.5826), from the mucus indicates that there is some presence of phospholipids in the mucus (Figure 1d, insert). Overall, we show that the metabolome of living ALI-cultured cells can be effectively investigated with nano-DESI-MS.

### 2.2. Transportation and Metabolism of Histamine and Corticosteroids

The combination of the living cells and nano-DESI MS is an easy-to-use tool for characterizing general membrane transport properties and in vivo monitoring of the metabolism of exogenous molecules. To investigate the basal–apical transport of histamine through the ALI lung tissue model, histamine was added to the medium on the basal side of the insert. Subsequently, the apical side was sampled and analyzed with nano-DESI-MS. In an experiment, four different concentrations of histamine were added to four different inserts that were sampled after 15 min of incubation. Despite the addition of histamine on the basal side of the cells, the signal was detected at the apical side after 15 min, showing that histamine is transported through the ALI lung epithelial tissue model. The histamine signal increased linearly with increasing concentration (Figure S1); this implies concentration-independent transport of the molecule through the living cell tissue model in the investigated concentration range.

**Figure 2:**
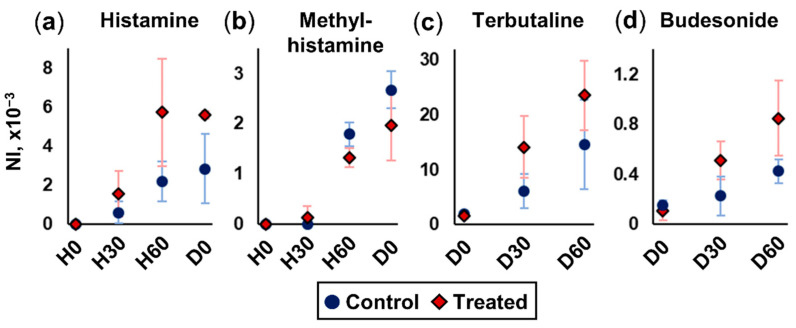
Differences in histamine and drug transportation attributed to different cell permeability between the Treated and the Control groups. (**a**) Transportation of histamine, (**b**) accumulation of methylhistamine after histamine exposure, (**c**) terbutaline transportation, and (**d**) budesonide transportation. Sampling was performed at 0, 30, and 60 min after exposure to histamine (H0, H30, H60, respectively) and at 0, 30, and 60 min after drug exposure (D0, D30, and D60, respectively). All intensity values are normalized to the total ion current (NI) at each scan event and presented as NI, ×10^3^ on the y axes. Error bars represent one standard deviation.

The analysis of living cells opens a new opportunity in metabolomics, which is to investigate real-time metabolism. To compare the impact of IL-13 treatment on the cells’ capability for metabolism, a time-course study was conducted to investigate histamine metabolism. In this study, 100 µM of histamine was added to the basal side medium of both Control and IL-13-treated (Treated hereinafter) cells. Subsequently, nano-DESI MS sampling was performed from the ALI apical side (the air surface) at 0, 30, and 60 min after exposure to histamine (H0, H30, H60, respectively). The resulting data show a linear increase in histamine intensity during the defined time course (Figure 2a). Additionally, the intensity of histamine was overall higher in the Treated group compared to the Control. Furthermore, since the nano-DESI MS analysis generates data of all detectable analytes, the data also included the main bronchial epithelium metabolite, methylhistamine. Methylhistamine was detected in both the Control and Treated cells 30 min after exposure to histamine, but only at low intensities. However, after 60 and 90 min (H60 and D0, respectively), it was clearly detected at a higher intensity in the Control cells compared to the Treated cells (Figure 2b). Overall, the detected intensities imply faster transportation and slightly slower metabolism for basally introduced histamine in the Treated cell layers.

After histamine exposure, the cells were exposed to the corticosteroids terbutaline and budesonide, which are commonly used for asthma treatment [34,35]. The short-time effect of these drugs was investigated on epithelium cells. The sampling was performed at 0, 30, and 60 min into drug exposure (D0, D30, and D60, respectively), where the 0 point was 90 min after exposure to histamine. Similar to the results for histamine, the drug was transported to a higher degree through the Treated cells compared to the Control cells, which correlates with the expected permeability of the cell layer being higher after IL-13 treatment [36] (Figure 2c,d). No metabolites of terbutaline (terbutaline sulfate and terbutaline glucuronide [37]) or budesonide (16 α-hydroxyprednisolone and 6 β-hydroxy-budesonide [38]) were detected, which is likely due to their low ionization efficiency in positive ionization mode along with the expected low abundance since their elimination half-times are several hours [39,40]. Follow-up studies with longer exposure times and negative ionization mode are necessary to further investigate this drug metabolism. During drug treatment, the intensity of histamine decreased. The levels of histamine decreased after 30 min in the Control group, and after 60 min in the Treated group (Figure S2a). However, the signals of methylhistamine remained even after 60 min of drug treatment (Figure S2b). To sum up, the results demonstrate the effectiveness of using nano-DESI MS for monitoring real-time transport and metabolism of exogenous molecules in living ALI cells.

### 2.3. Metabolome Alterations in Asthma and Drug Treatment

#### 2.3.1. Non-Targeted Overview of Endogenous Metabolism

**Figure 3:**
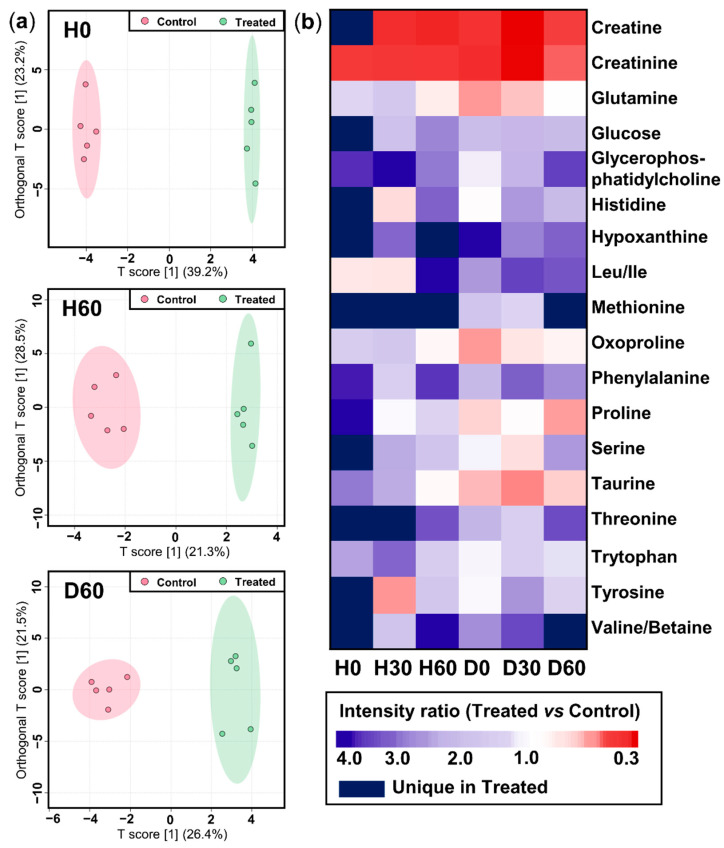
Non-targeted endogenous metabolite profiling of the living cells in the Treated and Control groups using nano-DESI MS. (**a**) OPLS-DA plots highlighting the general metabolome difference between the groups before histamine and drug treatment (H0), after the histamine exposure (H60), and after drug treatment (D60). (**b**) Heatmap showing the intensity ratios of metabolites between the Treated and Control sample groups at six time points over the course of treatments by histamine (H0) and drug (D0). Only metabolites reliably annotated as sodium adducts are presented, data corresponding to all annotated features are shown in Table S1. Red color represents upregulation and blue color represents downregulation in the Treated group. All intensity values were normalized to the total ion current prior to analysis.

The non-targeted analysis approach of nano-DESI MS enables general profiling of endogenous metabolome alterations in living cells during histamine exposure and drug treatment. The time points and respective conditions of the experiments were the same as described in Section 2.2. The metabolite profiles of the Control and Treated groups were clearly distinct, forming two separate groups on the orthogonal projection to latent structure discriminant analysis (OPLS-DA) plots in their original state (H0), after histamine exposure (H60), and after drug treatment (D60) (Figure 3a). This distinction between the two conditions was also corroborated by the detected intensities of the individual endogenous metabolites. In particular, 39 features (H^+^, Na^+^, or K^+^ adducts) were detected and subsequently annotated as 26 different endogenous metabolites using accurate mass (Table S1).

Compared to the Control, the Treated group behaved metabolically differently upon histamine exposure and drug treatment; this could be monitored using the instrumental setup described above. We illustrate the alterations of 18 metabolites on a heatmap over the six different time points (Figure 2b). This displays the intensity ratios (Treated over Control) of sodium adducts of the annotated metabolites detected at a minimum of two different time points. Creatine and creatinine were decreased in the Treated sample group at all time points, while glucose, hypoxanthine, phenylalanine, and valine were increased in the Treated group. The unique time-resolved in situ data further showed a shift in metabolite abundances due to treatments. For example, proline, oxoproline, and taurine showed an increase during histamine exposure (H0–H60) and a subsequent decrease during drug treatment (D0–D60) in the Treated sample group.

#### 2.3.2. Arginine and Glutamine Metabolism

**Figure 4:**
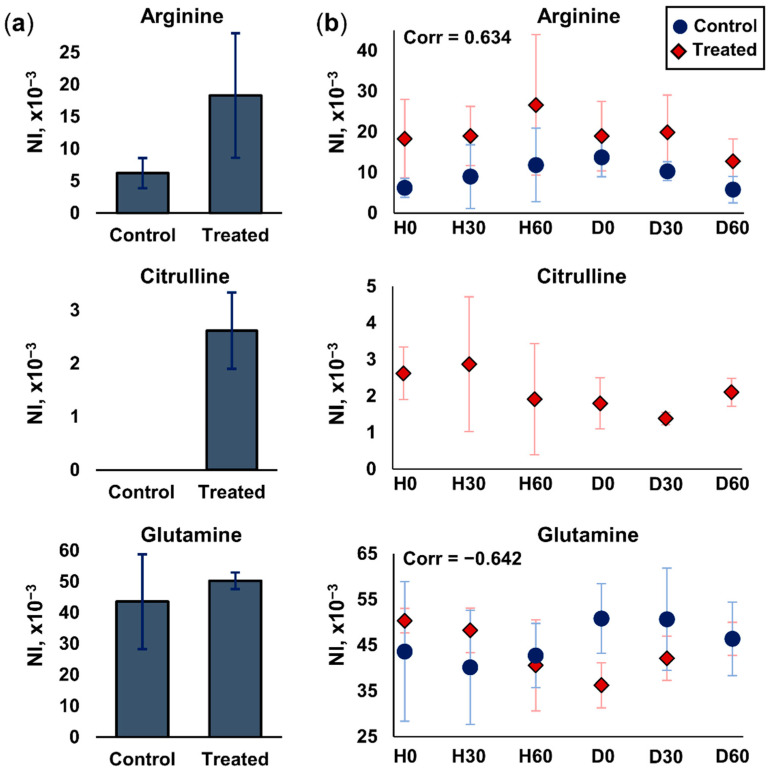
Differences in detected intensities of targeted endogenous metabolites between the Treated and Control cells. (**a**) Arginine, citrulline, and glutamine levels before histamine and drug exposure (H0 timepoint). (**b**) Arginine, citrulline, and glutamine levels throughout the time course of histamine exposure and drug treatment. Citrulline was never detected in any of the Control samples. Arginine was detected as a protonated adduct, while citrulline and glutamine are presented as sodiated adducts. Sampling was performed at 0, 30, and 60 min after exposure to histamine (H0, H30, H60, respectively) and at 0, 30, and 60 min after drug exposure (D0, D30, and D60, respectively). All intensity values are normalized to the total ion current (NI) at each scan event. Error bars represent one standard deviation. Pearson correlation coefficients are displayed on the graphs for arginine and glutamine to show the difference in cellular behavior of the Control and Treated cells during the time of the investigation.

In the IL-13-induced asthma model, the most affected pathways were found to be arginine and glutamine metabolism. Specifically, the in situ nano-DESI analysis of the living ALI lung cell model revealed a signal increase of arginine in the Treated group as compared to the Control (Figure 4a). Furthermore, citrulline was only detected in the samples of the Treated group, while the glutamine signal was found to be constant between the groups and therefore not affected by IL-13 treatment (Figure 4a).

Interestingly, the exposure to histamine and drugs was found to directly impact the detected abundance of these metabolites. During the time course of histamine exposure, the intensity of arginine slightly increased followed by a decrease during drug treatment in both the Control and Treated groups (Figure 4b). The intensity of citrulline in the Treated group showed opposite trends to arginine during histamine exposure and drug treatment, with a decrease followed by an increase (Figure 4b). Despite treatment, citrulline was never detected in the Control group. The IL-13 treatment also had a remarkable effect on the intensity of glutamine upon exposure. Despite being similar at time zero, histamine exposure caused a decrease in the Control cells and an increase in the Treated cells, which was followed by an increase in the Control cells and a decrease in the Treated cells during drug exposure. This behavior showed a strong negative correlation (Figure 4b). Overall, the differential metabolome behavior during a time course of treatments of ALI lung epithelium cells compared to IL-13 treatment was successfully monitored in vivo.

### 2.4. Proteomics Profiling after Histamine and Drug Treatment

**Figure 5:**
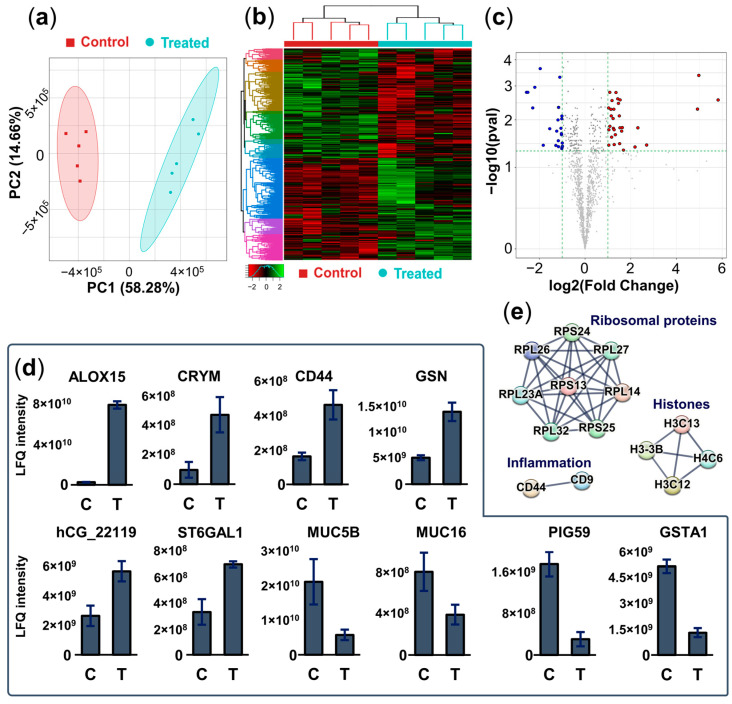
Differences in the proteomic profile of the Treated and the Control sample groups (for analysis details, see Section 4.5.2). (**a**) Principal component analysis of the samples analyzed based on proteins annotated in both groups. (**b**) Hierarchical clustering heatmap on all quantified proteins (protein LFQ intensity values were Z-scored). The histogram in the bottom left corner shows the correspondence between color hues and values. (**c**) Volcano plot of all quantified proteins. The vertical dashed lines denote a 2-fold change while the horizontal dashed line denotes *p* = 0.05 significance threshold. Blue—significantly lower in the Treated sample group, Red—significantly higher in the Treated sample group. The detailed list of significantly altered proteins can be seen in Tables S2 and S3 and Figure S3. (**d**) MaxQuant LFQ intensities of selected proteins showing significantly different quantities (FC > 2 or FC < 0.5, Benjamini-Hochberg corrected *p*-value < 0.05) in the two groups. Proteins are denoted with corresponding gene names; error bars represent the standard deviation in each group. C: Control, T: Treated. (**e**) Protein interaction network built on the significantly upregulated proteins in the Treated group with the STRING webserver [41]. High confidence (0.7) interactions are shown, the disconnected nodes are hidden, and all other settings are set as default.

After the histamine exposure and drug treatment, the cells were flash frozen for subsequent proteomics analysis to discover differences in the protein expression level upon IL-13 treatment of lung epithelial cells. The cell layer was subjected to digestion on the surface and the resulting peptide mixtures were subsequently analyzed by single-shot nanoUHPLC-MS/MS measurements. We identified 1164–1336 proteins in the Control samples, and 1231–1396 proteins in the Treated samples. Proteins identified in at least three samples in both groups were then selected for quantitative comparison; in total, 1355 proteins were quantified. Using principal component analysis (PCA), the data for the Control and Treated groups were well-separated, mainly by the first principal component (Figure 5a), which is also true for the heatmap based on hierarchical clustering (Figure 5b). This implies that there were considerable molecular differences in the proteomics level between the two groups.

To identify the proteins responsible for this differentiation, pairwise comparison tests were performed between the Control and Treated sample groups (for details see Section 4). Based on the results, 31 proteins were found to be significantly upregulated (FC > 2, *p* < 0.05), and 22 to be significantly downregulated (FC < 0.5, *p* < 0.05) in the Treated sample group (Figure 5c). For the detailed lists of differentially expressed proteins, see Tables S2 and S3 and Figure S3. Examples of significantly upregulated and downregulated proteins are shown in Figure 5d. Proteins with oxidation–reduction activity (e.g., Arachidonate 15 lipoxygenase, ketimine reductase), inflammatory proteins (CD44 antigen and Gelsolin), and other proteins involved in glycosylation (Galectin and Beta galactoside alpha-2,6 sialyltransferase 1) were upregulated. On the other hand, proteins mainly involved in extracellular matrix formation (e.g., Mucins 5B and 16) were significantly downregulated, along with glutamine synthase and Glutathione S transferase A1. Identification of the dysregulated pathways in the Treated group was achieved using a protein interaction network built on the significantly upregulated proteins using the STRING webserver (Figure 5e). Three overrepresented pathways were observed: ribosomal proteins, histones, and inflammatory molecules. Thus, functionalities related to inflammatory response and subsequent protein synthesis are strongly involved in the molecular differences related to the IL-13 treatment of lung epithelial cells.

Some proteins were identified as uniquely detected in either the Control or Treated group, which means that they are strongly over- or under-expressed in the respective group, but the degree of changes cannot be quantified. Specifically, proteins were considered “unique” to a group if they were detected in all five samples in one group and none of the samples in the other. Overall, there were 22 and 4 proteins that were found to be unique in the Treated and the Control groups, respectively (Table S4). The two uniquely detected proteins with the highest confidence of annotation (i.e., the highest number of unique peptides) were Nitric oxide synthase and Serpin B2, both known to be involved in asthma development. Overall, the IL-13 treatment of lung epithelial cells cultured using ALI displays proteome remodeling that is mainly connected to the inflammatory response and extracellular signaling.

## 3. Discussion

We performed a time-resolved in vivo nano-DESI-MS investigation of living lung cells in an air–liquid interface culture under different conditions and treatments with a focus on secreted small molecule metabolites. Metabolic changes specifically connected to IL-13-related inflammation were monitored in real-time by sampling the living cells. Subsequently, a “bottom-up” proteomics analysis was performed where the cellular layer was digested directly on the surface of the ALI wells. Overall, we identified dynamic shifts in metabolome and proteome profiles after IL-13 treatment and metabolome changes after relatively short exposures to histamine and drugs, reflecting the fast turnover of the asthmatic model system.

Nano-DESI is an emerging ambient ionization technique operating with localized liquid extraction and subsequent nanoESI ionization. The main advantage of nano-DESI sampling is the elimination of sample preparation prior to analysis, which enables living cells to be measured. This not only simplifies the workflow and enables in vivo measurements but suggests minimal spontaneous and enzymatic molecular degradation since molecules are detected seconds after sampling. Additionally, the diverse set of detected molecules reflects the actual chemical environment of the sampled cell layer. The use of the ALI—nano-DESI MS setup conveniently permits the basal treatment of the cell layer with different molecules one by one or right after each other to study instantaneous metabolome shifts. This opens a novel way of designing metabolomics studies.

The sampling was performed at ca. 100 µm diameter spots, which implies minimal local damage to the cell layer and therefore a conserved cellular metabolism. We observed that nano-DESI MS was an effective tool for temporarily resolved in vivo study of cell metabolism. However, some limitations include the fact that sampling is performed merely on the viscous mucus layer, which includes denatured and native (glyco)proteins that may clog the nano-DESI capillaries and cause signal drops. Therefore, a future optimization of the extraction solvent is planned as well as the use of pneumatically-assisted nano-DESI MS [42].

In this study, we demonstrated an analysis with nano-DESI MS for determining the membrane transport characteristics of a living cell layer for the first time. The generally utilized methods for studying transcellular transport involve the use of transwell inserts, i.e., immersing both sides of the cellular layer into media [43], which would suffocate the lung epithelium cells. The combination of ALI cultured cells and nano-DESI MS, therefore, provides an invaluable alternative that might be essential in drug development for determining drug target concentration on the apical side of the lung tissue. First, we performed a concentration-dependent study of histamine transportation and determined that the transportation of histamine through the cellular layer was concentration-independent. Then, we performed temporally resolved monitoring of histamine and corticosteroids; we observed faster transportation in the IL-13-treated group for all the molecules. This finding is in agreement with the previous literature findings that the permeability of asthmatic cells was higher than that of healthy cells because of epithelial barrier disruption, which makes the epithelium more susceptible to environmental exposure [36,44]. Moreover, the proteomics investigation revealed the downregulation of various cellular connectivity proteins (such as Transmembrane channel-like protein 1), further corroborating differential membrane transport characteristics under asthmatic conditions. Contrary to larger levels of histamine uptake in IL-13-treated cells, we observed slower metabolism both with and without the addition of asthma drugs. This may be attributed to the decreased activity of the enzyme Histamine *N*-methyltransferase (HNMT), which is responsible for histamine’s metabolomic transformation in the bronchial epithelium [45]. The differential abundance of HNMT, however, was not reflected in the proteomics analysis. Therefore, we note that the high mucus secretion and enhanced permeability of Treated cells may result in increased histamine localization in the mucus, thus restricting its metabolism by HNMT, which is mainly localized in the cytoplasm [46]. Further studies are thus proposed on HNMT activity in ALI lung cells.

Then, we characterized the endogenous metabolome and the cellular proteome in connection with IL-13-treated cells; the two sample groups were well-distinguished based on both molecular families. The nano-DESI MS analysis permitted the in vivo annotation of 26 endogenous small metabolites; most of them were validated through the formation of several adduct forms. The intensity of arginine showed a slight increase during histamine exposure, a slight decrease during drug treatment in both sample groups, and a consistently higher abundance in the Treated group throughout the study. This is in agreement with previous findings that a higher arginine content is related to asthmatic behavior [47]. Citrulline was only detected in the Treated group, its levels showed a minor decrease by the end of histamine treatment. Budesonide treatment had been found to inhibit the expression of iNOS and, therefore, the synthesis of citrulline [48], however, no further decrease in citrulline levels was observed during the time course of the drug exposure. The “bottom-up” proteomics analysis after the drug treatment showed that the iNOS was only detected in the Treated group, hence the unique detection of citrulline is explained by the proteomic profiling.

The glutamine metabolism showed opposite trends of regulation in the two sample groups of the Control and IL-13-treated ALI cells. Glutamine substantially decreased in the IL-13-treated cells after the histamine exposure. The enzyme glutamine synthetase, which is responsible for glutamine synthesis and metabolism, was downregulated in the Treated cells. High concentrations of IL-13 and histamine might cause inflammation. As a result, reactive oxygen species (ROS) and reactive nitrogen species (RNS) are produced in greater amounts, including nitric oxide (NO) [49]. Thus, glutamine synthetase can be considerably inactivated due to oxidative stress [50]. These processes explain the decrease in glutamine during histamine exposure, which causes inflammation in epithelium cells. It was recently found that glutamine deficiency causes neutrophilic asthmatic inflammation [51], making it worthy of consideration for future studies of inflammatory asthma responses. Other amino acids contributing to the differentiation of the Control and Treated sample groups have also been shown to differentiate between different etiologies and severities of asthma. For example, glutamine and valine plasma levels were found to differentiate between mildly and highly sensitized childhood asthma [52], while plasma levels of methionine, arginine, carnitine, and lysine were correlated with exacerbation-prone childhood asthma [53].

After histamine exposure, both groups were treated with terbutaline and budesonide corticosteroids for 60 min. On the metabolomic level, this caused differences in the regulation of a handful of amino acids, such as leucin/isoleucine, proline, oxoproline, serine, and taurine. While the intensity of these molecules showed an increase during histamine exposure (H0–H60), there was a substantial decrease during corticosteroid treatment (D0–D60) in the Treated sample group. This indicates their importance in a sensitive response to allergic attacks and drug treatment in IL-13-treated lung epithelial cells. We also identified three highly upregulated proteins (with fold changes of over 25), FCGBP, ALOX15, and SERPINB2, which play critical roles in human allergy, immune responses, and asthma [54,55,56]. The roles of increased ribosomal activity and histones were strongly reflected in our studies, as the proteomics pathways shown by the upregulated proteins in the Treated group revealed highly enriched pathway activity. Both of these pathways have already been shown to be involved in asthma pathology [57,58]. The activation of the NF-κB signaling pathway is also reflected in our results via the upregulation of Ribosomal protein S3 [59].

Glycosylation undergoes a major remodeling during asthmatic behavior. We observed the upregulation of the ST6GAL1 sialyltransferase along with the metabolomic downregulation of one *N*-acetylneuraminic acid derivative during drug treatment (it was only detected in the Control group). As sialylation of mucins is associated with type-2 inflammation [60], it is hypothesized to be a consequence of specifically inducing asthmatic behavior with IL-13. Along with the already well-associated Mucin 5B downregulation [61], we found that Mucin 16 was downregulated in the Treated sample group as well. These results suggest that the expression of mucins decreases in inflammatory asthma along with the rate of sialylation. The distribution of mucins has already been shown to substantially regulate the (glyco)proteomic profile of lung tissues in other diseases, such as lung cancer, as well [62]. Galectins, a protein family that binds to β-galactosides, were downregulated, while the Galectin-3-binding protein was non-significantly upregulated. As a result, we propose the importance of carrying out site-specific glycosylation studies to determine the exact glycan compositional changes accompanying IL-13-induced asthma.

## 4. Materials and Methods

### 4.1. Reagents and Standards

For the nano-DESI solvent, HPLC-MS grade methanol (BDH Chromanorm Solvents, VWR International AB, Stockholm, Sweden), deionized water (18.2 MΩ, Milli-Q, Millipore, Solna, Sweden), and formic acid (98-100%, Merck, Darmstadt, Germany) were used. Interleukine-13 and histamine were purchased (Sigma-Aldrich, Stockholm, Sweden), and budesonide and terbutaline were extracted from commercially available products. The medium used for growing the cells consisted of 50 mL of Dulbecco’s Modified Eagle Medium (500 mL of D-MDM, 5 mL of 100 mM Minimum Essential Medium Sodium pyruvate (Gibco, MEM NaPyr, Fisher Scientific, Gothenburg, Sweden), 5 mL of 200 mM L-Glutamine, 5 mL of 100× Minimum Essential Medium Non-Essential Amino Acids (Gibco MEM NEA, Fisher Scientific, Gothenburg, Sweden), and 50 mL ALI medium (250 mL of Bronchial Epithelial Cell Growth Basal Medium (BEBM, Lonza Bioscience, Stockholm, Sweden), 1 vial of Bronchial Epithelial SingleQuots Kit (BEGM, Lonza Bioscience, Stockholm, Sweden), and 500 µL 1.5 mg/mL BSA), with an addition of 50 µL 0.1 mM retinoic acid. All the other reagents were purchased from Thermo Fisher, Waltham, MA, USA.

### 4.2. Cell Culturing and Air–Liquid Interface (ALI) Model

For an in vitro model of the epithelium layer with living cells, an air–liquid interface (ALI) was used as it is described elsewhere [13]. The second passage of clonal primary human bronchial epithelial cells was placed on semipermeable membranes in two six-well plates and submerged in the freshly prepared medium for four days until they were one hundred percent confluent. When confluent, the medium was removed from the apical side of the cells allowing the cells to be cultured in ALI. Approximately one week after differentiation, the goblet cells started to produce mucus. The cells were left in this configuration for thirty days prior to any experiments being performed, and the mucus was continuously removed every two days by suction to keep the cells healthy. Then, the “Treated” sample group was exposed to interleukine-13 (IL-13) at 10 ng/mL for 5 days (medium changed every other day), while the “Control” group was incubated with the IL-13-free medium for the same timeframe.

### 4.3. Metabolomics Analysis

#### 4.3.1. Instrumentation

The nano-DESI setup was used to analyze the metabolome of the living ALI-cultured cells, with slight modifications to previously described setups [31]. Briefly, two fused silica capillaries (50 μm ID and 150 μm OD, GENETEK, Gothenburg, Sweden) were positioned together, and the secondary capillary was bent about 45° to allow it to reach the cellular layer in the ALI wells. A liquid bridge between the two capillaries was formed by delivering the extraction solvent (MeOH:H_2_O *v*/*v* 9:1) at 0.5 µL/min using a syringe pump. The molecules were extracted directly from one unique spot per analysis time point in a continuous flow of the solvent and delivered into the mass spectrometer through electrospray ionization. The experiments were performed on a Velos Orbitrap (ThermoFisher, Waltham, MA, USA) at a resolution of 60,000 (m/Δm at *m*/*z* 200), a heated capillary temperature of 250 °C, and an ESI voltage of 3 kV. The AGC target was set to 1 × 10^6^, and mass spectra were recorded in the *m*/*z* 100–1000 range.

#### 4.3.2. Time-Resolved Treatment Investigation of ALI Cell Surface

To analyze the model lung tissue, one insert was removed from the six-well plate and placed on a glass slide with a small drop of medium between the glass and the insert. Three parallel touchdown measurements were carried out at each condition. When the solvent junction of the nano-DESI probe came into contact with the sample, molecules mostly from the mucus layer were desorbed and ionized by electrospray at the inlet of the mass spectrometer. For both the IL-13-treated and Control groups, five samples each (5 + 5 individual transwell cultures) were analyzed. To study temporally-resolved drug transportation and subsequent metabolome alterations, the cells were treated with 100 µM of histamine and analyzed at 0, 30, and 60 min time points. After this, the anti-asthmatic drugs terbutaline (100 µM) and budesonide (300 µM) were added to the medium, and sampling was carried out at 0, 30, and 60 min after drug exposure. After nano-DESI MS analysis, all inserts with the ALI cells were snap-frozen and stored at –80 °C prior to proteomics analysis.

### 4.4. Proteomics Analysis

#### 4.4.1. Sample Preparation

Cell lysis and protein extraction were performed in a buffer containing 20 nM HEPES, 9 M urea, and a Complete Mini-EDTA-free protease cocktail (Roche, Solna, Sweden) aided by sonication with a probe (10 s, 3 mm probe, pulse 1 sec, amplitude 30%). The samples were centrifuged afterward, and the supernatant was used for protein analysis. The total protein concentration in each sample was measured using a Coomassie assay, with bovine serum albumin as a standard (BioRad, Solna, Sweden). Aliquots corresponding to 20 μg protein were reduced with dithiothreitol (DTT, Sigma Aldrich, working concentration 50 mM) for 15 min at 50 °C and alkylated with iodoacetamide (IAA, Sigma Aldrich, working concentration 25 mM) for 15 min at room temperature in the dark. After two times dilutions with 50 mM ammonium bicarbonate, trypsin (Promega, Madison, WI, USA) was added at a trypsin:protein ratio of 1:20, and digestion was performed overnight at 37 °C. Digestion was stopped by adding 35 µL (1/4 of total volume) of 2% trifluoroacetic acid (TFA), 20% acetonitrile (ACN), and 78% water. After digestion, the samples were dried down and purified using Pierce C_18_ Spin Columns (Thermo Scientific, USA) applying the manufacturer’s protocol. After elution, peptides were vacuum centrifuged to dryness using a Speedvac system ISS110 (Thermo Scientific) and stored at −80 °C until further use.

#### 4.4.2. NanoHPLC-MS/MS Analysis

LC-MS/MS analysis for proteomics was performed using an EASY-nLC 1000 system (Thermo Fisher Scientific) coupled to a QExactive Plus mass spectrometer (Thermo Fischer Scientific) equipped with a nano-ESI source. Solvent A was 0.1% formic acid (FA) in H_2_O and solvent B was 0.1% FA in acetonitrile (ACN). Peptides were reconstituted in solvent A to a nominal concentration of 0.2 μg/μL, and 5 μL (1 μg) from each sample was injected onto a C_18_ pre-column (75 µm i.d., 2 cm length, 3 µm particle size, Thermo Fischer Scientific). Peptide separation was carried out using a linear 150 min gradient from 4 to 100% B with a flow rate of 250 nL/min through the pre-column and the following analytical C_18_ column (75 µm i.d., 15 cm length, 2 µm particle size, Thermo Fischer Scientific). The mass spectrometer was operated in positive mode with an electrospray voltage of 2.2 kV. The survey MS spectra (400–1750 *m*/*z*) were generated at a resolution of 70,000 (FWHM) using an automatic gain control (AGC) target of 3 × 10^6^. Data were recorded in data-dependent mode, where the top ten most abundant peaks were selected for fragmentation using higher energy collision-induced fragmentation with nitrogen. A normalized collision energy of 25% was applied. The AGC target for MS/MS was set to 5 × 10^5^ at a resolution of 17,500. Dynamic exclusion was used with an exclusion period of 20 s.

### 4.5. Data Analysis

#### 4.5.1. Metabolomics

Mass spectra were analyzed in ThermoFisher Xcalibur QualBrowser. Thermo RAW files were converted into centroid mzXML files using MSConvertGUI v3.0 (ProteoWizard) and further analyzed in Matlab R2022b. The first 20 scans were considered for analysis from each sampling spot. The absolute peak intensities were extracted for *m/z* of interest (5 ppm tolerance) and each intensity was normalized to the scan total ion current and then averaged for the 20 scans, excluding 0 intensity values. The detected features were filtered for two group comparisons; only those that were quantified in at least two samples of both sample groups were considered. Molecules were considered uniquely detected in one group (Control or Treated) when they were detected in at least 60% of the samples in one group and at most 20% in the other group at a given condition (timepoint). OPLS-DA plots were created using Metaboanalyst 5.0 to compare the metabolomic profiles of the Treated and Control groups. All adducts were used for all annotated metabolite features, and missing values were imputed with LoDs (1/5 of the minimum positive value of each variable).

#### 4.5.2. Proteomics

The acquired data (RAW files) were processed by MaxQuant software, version 1.5.3.30 [63], and database searches were performed using the implemented Andromeda search engine [64]. MS/MS spectra were correlated to a FASTA database containing proteins from Homo sapiens extracted from the UniProt database (UP000005640, accession date: 15 June 2016). A decoy search database, including common contaminants and a reverse database, was used to estimate the identification false-discovery rate, where a rate of 1% was accepted. The following settings were used in the search: minimum peptide length of seven amino acids, 20 ppm mass tolerance in the first search, 4.5 ppm mass tolerance in the second search, 20 ppm mass tolerance for the fragment ions, trypsin as digestive enzyme, and two maximum missed cleavages were allowed. Carbamidomethylation was set as a fixed modification and the variable modifications were Oxidation (M) and Deamidation (NQ). The search criteria for protein identification were set to at least two matching unique peptides. Proteins were considered uniquely detected if they were identified in all five samples of one group and none of the samples in the other group, and were disregarded in the quantitative comparisons. Proteins present in at least 60% (three out of five) of the samples in both groups were selected for the quantitative comparison of the two groups. Data analysis was based on label-free quantification (LFQ) intensities for the proteins. Statistical analysis was performed with the BioStatFlow webserver (v2.9, http://www.biostatflow.org, accession date: 15 February 2023) [65]. Data were log2-transformed, and the imputation of missing data points was performed using the kNN algorithm (k = 10). Principle component analysis was performed; ellipses represent a confidence level of 0.95. Hierarchical clustering was performed based on Euclidian distances; variables were reduced to 75% of the original using the Kruskal–Wallis test and Benjamini–Hochberg FDR correction. For the quantitative comparison of the two groups, the Wilcoxon *t*-test was performed; false discovery rates were controlled using the Benjamini–Hochberg method at 5%. The fold change (FC) was defined by the ratio FC = (LFQ_Treated_/LFQ_Control_). When defining upregulated and downregulated proteins in the Treated group compared to the Control group, FC > 2 (upregulated) or FC < 0.5 (downregulated) were defined as the criteria. A gene enrichment analysis was performed on the proteins that were upregulated in the Treated group with the STRING webserver (v12.0, https://string-db.org/, accession date: 5 February 2024) [41] using high confidence (0.7) interactions and all other settings set as default.

## 5. Conclusions

In this study, we used nano-DESI MS to characterize the cellular metabolome followed by “bottom-up” proteomics. We demonstrated the feasibility of in vivo nano-DESI-MS measurements that allowed for the real-time measurement of transport and metabolism in the ALI lung-cell layer. Nano-DESI MS proved to be an efficient tool for measuring the permeability differences in the cell layers as well as characterizing the cellular metabolome. Additionally, the metabolomic remodeling was successfully monitored in connection with the histamine and corticosteroid treatment of the IL-13-induced asthma model. The subsequent proteomics measurements showed a good correlation with the metabolomic alterations and the previous literature findings along with unraveling new (glyco)proteomic targets for asthma treatment. This implies the unbiased utility of in vivo nano-DESI for characterizing ALI tissue layers.

## Data Availability

Original mass spectrometry data files have been deposited in the MassIVE database under the accession number MSV000091751, and can be downloaded via FTP (ftp://massive.ucsd.edu/MSV000091751/, accessed on 22 February 2024).

## Supplementary Materials

The following supporting information can be downloaded at the following location: https://www.mdpi.com/article/10.3390/ijms25095034/s1.

## References

[1] Soriano J.B., Kendrick P.J., Paulson K.R., Gupta V., Abrams E.M., Adedoyin R.A., Adhikari T.B., Advani S.M., Agrawal A., Ahmadian E. (2020). Prevalence and attributable health burden of chronic respiratory diseases, 1990–2017: A systematic analysis for the Global Burden of Disease Study 2017. Lancet Respir Med..

[2] Wenzel S.E. (2013). Complex phenotypes in asthma: Current definitions. Pulm. Pharmacol. Ther..

[3] Vatrella A., Fabozzi I., Calabrese C., Maselli R., Pelaia G. (2014). Dupilumab: A novel treatment for asthma. J. Asthma Allergy.

[4] Wills-Karp M. (2004). Interleukin-13 in Asthma Pathogenesis. Immunol. Rev..

[5] Yamauchi K., Ogasawara M. (2019). The Role of Histamine in the Pathophysiology of Asthma and the Clinical Efficacy of Antihistamines in Asthma Therapy. Int. J. Mol. Sci..

[6] Corren J. (2013). Role of Interleukin-13 in Asthma. Curr. Allergy Asthma. Rep..

[7] Robin L. Thurmond. Histamine in Inflammation. (2010). https://link.springer.com/book/10.1007/978-1-4419-8056-4.

[8] Laitinen L.A., Laitinen A., Haahtela T. (1992). A comparative study of the effects of an inhaled corticosteroid, budesonide, and a β2-agonist, terbutaline, on airway inflammation in newly diagnosed asthma: A randomized, double-blind, parallel-group controlled trial. J. Allergy Clin. Immunol..

[9] Alangari A.A. (2014). Corticosteroids in the treatment of acute asthma. Ann. Thorac. Med..

[10] Hanratty C.E., Matthews J.G., Arron J.R., Choy D.F., Pavord I.D., Bradding P., Brightling C.E., Chaudhuri R., Cowan D.C., Djukanovic R. (2018). A randomised pragmatic trial of corticosteroid optimization in severe asthma using a composite biomarker algorithm to adjust corticosteroid dose versus standard care: Study protocol for a randomised trial. Trials.

[11] Liu Y., Zhang S., Li D., Jiang S. (2013). Efficacy of Anti-Interleukin-5 Therapy with Mepolizumab in Patients with Asthma: A Meta-Analysis of Randomized Placebo-Controlled Trials. PLoS ONE.

[12] Clarus Leung X., Wadsworth S.J., Jasemine Yang S., Dorscheid D.R. (2020). Structural and functional variations in human bronchial epithelial cells cultured in air-liquid interface using different growth media. Am. J. Physiol. Lung Cell Mol. Physiol..

[13] Chen S., Schoen J. (2019). Air-liquid interface cell culture: From airway epithelium to the female reproductive tract. Reprod. Domest. Anim..

[14] Baldassi D., Gabold B., Merkel O.M. (2021). Air−Liquid Interface Cultures of the Healthy and Diseased Human Respiratory Tract: Promises, Challenges, and Future Directions. Adv. NanoBiomed Res..

[15] Jiang D., Schaefer N., Chu H.W. (2018). Air–Liquid Interface Culture of Human and Mouse Airway Epithelial Cells. Lung Innate Immunity and Inflammation.

[16] Ghio A.J., Dailey L.A., Soukup J.M., Stonehuerner J., Richards J.H., Devlin R.B. (2013). Growth of human bronchial epithelial cells at an air-liquid interface alters the response to particle exposure. Part. Fibre Toxicol..

[17] Everman J.L., Rios C., Seibold M.A. (2018). Utilization of Air–Liquid Interface Cultures as an In Vitro Model to Assess Primary Airway Epithelial Cell Responses to the Type 2 Cytokine Interleukin-13. Type 2 Immunity: Methods and Protocols.

[18] Schmidt H., Braubach P., Schilpp C., Lochbaum R., Neuland K., Thompson K., Jonigk D., Frick M., Dietl P., Wittekindt O.H. (2019). IL-13 Impairs Tight Junctions in Airway Epithelia. Int. J. Mol. Sci..

[19] Seibold M.A. (2018). Interleukin-13 Stimulation Reveals the Cellular and Functional Plasticity of the Airway Epithelium. Ann. Am. Thorac. Soc..

[20] Khalil S.M., Bernstein I., Kulaga H., Gour N., Rowan N., Lajoie S., Lane A.P. (2020). Interleukin 13 (IL-13) alters hypoxia-associated genes and upregulates CD73. Int. Forum Allergy Rhinol..

[21] Kotas M.E., Moore C.M., Gurrola J.G., Pletcher S.D., Goldberg A.N., Alvarez R., Yamato S., Bratcher P.E., Shaughnessy C.A., Zeitlin P.L. (2022). IL-13–programmed airway tuft cells produce PGE2, which promotes CFTR-dependent mucociliary function. JCI Insight.

[22] Rinschen M.M., Ivanisevic J., Giera M., Siuzdak G. (2019). Identification of bioactive metabolites using activity metabolomics. Nat. Rev. Mol. Cell Biol..

[23] Kelly R.S., Dahlin A., McGeachie M.J., Qiu W., Sordillo J., Wan E.S., Wu A.C., Lasky-Su J. (2017). Asthma Metabolomics and the Potential for Integrative Omics in Research and the Clinic. Chest.

[24] Xu S., Panettieri R.A., Jude J. (2022). Metabolomics in asthma: A platform for discovery. Mol. Asp. Med..

[25] Aug A., Altraja S., Kilk K., Porosk R., Soomets U., Altraja A. (2015). E-Cigarette Affects the Metabolome of Primary Normal Human Bronchial Epithelial Cells. PLoS ONE.

[26] López-Rodríguez J.C., Rodríguez-Coira J., Benedé S., Barbas C., Barber D., Villalba M.T., Escribese M.M., Villaseñor A., Batanero E. (2021). Comparative metabolomics analysis of bronchial epithelium during barrier establishment after allergen exposure. Clin. Transl. Allergy.

[27] Stollmeier M., Kahlert S., Zuschratter W., Oster M., Wimmers K., Isermann B., Rothkötter H.-J. (2023). Air–liquid interface cultures trigger a metabolic shift in intestinal epithelial cells (IPEC-1). Histochem Cell Biol..

[28] Xu W., Janocha A.J., Leahy R.A., Klatte R., Dudzinski D., Mavrakis L.A., Comhair S.A.A., Lauer M.E., Cotton C.U., Erzurum S.C. (2014). A novel method for pulmonary research: Assessment of bioenergetic function at the air–liquid interface. Redox Biol..

[29] Meissner F., Geddes-McAlister J., Mann M., Bantscheff M. (2022). The emerging role of mass spectrometry-based proteomics in drug discovery. Nat. Rev. Drug Discov..

[30] Sun Y.V., Hu Y.-J. (2016). Integrative Analysis of Multi-omics Data for Discovery and Functional Studies of Complex Human Diseases. Adv. Genet..

[31] Cardoso-Palacios C., Lanekoff I. (2016). Direct Analysis of Pharmaceutical Drugs Using Nano-DESI MS. J. Anal. Methods Chem..

[32] Bergman H.M., Lanekoff I. (2017). Profiling and quantifying endogenous molecules in single cells using nano-DESI MS. Analyst.

[33] Brown N.A., Bron A.J. (1987). An Estimate of the Human Lens Epithelial Cell Size in vivo. Exp. Eye Res..

[34] Mansur A.H., Afridi L., Sullivan J., Ayres J.G., Wilson D. (2014). Continuous terbutaline infusion in severe asthma in adults: A retrospective study of long-term efficacy and safety. J. Asthma.

[35] Rimmer C., Hetelekides S., Eliseeva S.I., Georas S.N., Veazey J.M. (2021). Budesonide promotes airway epithelial barrier integrity following double-stranded RNA challenge. PLoS ONE.

[36] Xiao C., Puddicombe S.M., Field S., Haywood J., Broughton-Head V., Puxeddu I., Haitchi H.M., Vernon-Wilson E., Sammut D., Bedke N. (2011). Defective epithelial barrier function in asthma. J. Allergy Clin. Immunol..

[37] Davies M., Peramuhendige P., King L., Golding M., Kotian A., Penney M., Shah S., Manevski N. (2020). Evaluation of in vitro models for assessment of human intestinal metabolism in drug discovery. Drug Metab. Dispos..

[38] Moore C.D., Roberts J.K., Orton C.R., Murai T., Fidler T.P., Reilly C.A., Ward R.M., Yost G.S. (2013). Metabolic pathways of inhaled glucocorticoids by the cyp3a enzymes. Drug Metab. Dispos..

[39] Donnelly R., Seale J.P. (2001). Clinical pharmacokinetics of inhaled budesonide. Clin. Pharmacokinet.

[40] Nyberg L. (1984). Pharmacokinetic parameters of terbutaline in healthy man. An overview. Eur. J. Respir Dis. Suppl..

[41] Szklarczyk D., Gable A.L., Lyon D., Junge A., Wyder S., Huerta-Cepas J., Simonovic M., Doncheva N.T., Morris J.H., Bork P. (2019). STRING v11: Protein–protein association networks with increased coverage, supporting functional discovery in genome-wide experimental datasets. Nucleic Acids Res..

[42] Duncan K.D., Bergman H.-M., Lanekoff I. (2017). A pneumatically assisted nanospray desorption electrospray ionization source for increased solvent versatility and enhanced metabolite detection from tissue. Analyst.

[43] Tavelin S., Gråsjö J., Taipalensuu J., Ocklind G., Artursson P., Wise C. (2002). Applications of Epithelial Cell Culture in Studies of Drug Transport. Epithelial Cell Culture Protocols.

[44] Georas S.N., Rezaee F. (2014). Epithelial barrier function: At the front line of asthma immunology and allergic airway inflammation. J. Allergy Clin. Immunol..

[45] Smith J.S., Hilibrand A.S., Skiba M.A., Dates A.N., Calvillo-Miranda V.G., Kruse A.C. Human Histamine N-Methyltransferase Pharmacogenetics: Common Genetic Polymorphisms That Alter Activity. http://www.molpharm.org.

[46] Ogasawara M., Yamauchi K., Satoh Y.I., Yamaji R., Inui K., Jonker J.W., Schinkel A.H., Maeyama K. (2006). Recent advances in molecular pharmacology of the histamine systems: Organic cation transporters as a histamine transporter and histamine metabolism. J. Pharmacol. Sci..

[47] Guo F.H., Comhair S.A., Zheng S., Dweik R.A., Eissa N.T., Thomassen M.J., Calhoun W., Erzurum S.C. (2000). Molecular Mechanisms of Increased Nitric Oxide (NO) in Asthma: Evidence for Transcriptional and Post-Translational Regulation of NO Synthesis. J. Immunol..

[48] Lim S.A.M., Jatakanon A., John M., Gilbey T., O’CONNOR B.J., Chung K.F., Barnes P.J. (1999). Effect of Inhaled Budesonide on Lung Function and Airway Inflammation Assessment by Various Inflammatory Markers in Mild Asthma. Am. J. Respir Crit. Care Med..

[49] Henricks P.A.J., Nijkamp F.P. (2001). Reactive oxygen species as mediators in asthma. Pulm. Pharmacol. Ther..

[50] Butterfield D.A., Hensley K., Cole P., Subramaniam R., Aksenov M., Aksenova M., Bummer P.M., Haley B.E., Carney J.M. (1997). Oxidatively Induced Structural Alteration of Glutamine Synthetase Assessed by Analysis of Spin Label Incorporation Kinetics: Relevance to Alzheimer’s Disease. J. Neurochem..

[51] Kim J.M., Im Y.N., Chung Y.J., Youm J.H., Im S.Y., Han M.K., Lee H.K. (2022). Glutamine deficiency shifts the asthmatic state toward neutrophilic airway inflammation. Allergy: Eur. J. Allergy Clin. Immunol..

[52] Chiu C.Y., Cheng M.L., Chiang M.H., Wang C.J., Tsai M.H., Lin G. (2021). Integrated metabolic and microbial analysis reveals host–microbial interactions in IgE-mediated childhood asthma. Sci. Rep..

[53] Cottrill K.A., Stephenson S.T., Mohammad A.F., Kim S.O., McCarty N.A., Kamaleswaran R., Fitzpatrick A.M., Chandler J.D. (2023). Exacerbation-prone pediatric asthma is associated with arginine, lysine, and methionine pathway alterations. J. Allergy Clin. Immunol..

[54] Woodruff P.G., Boushey H.A., Dolganov G.M., Barker C.S., Yang Y.H., Donnelly S., Ellwanger A., Sidhu S.S., Dao-Pick T.P., Pantoja C. (2007). Genome-wide profiling identifies epithelial cell genes associated with asthma and with treatment response to corticosteroids. Proc. Natl. Acad. Sci. USA.

[55] Boyce J.A. (2022). The role of 15 lipoxygenase 1 in asthma comes into focus. J. Clin. Investig..

[56] Wu J., Lin R., Huang J., Guan W., Oetting W.S., Sriramarao P., Blumenthal M.N. (2014). Functional Fcgamma Receptor Polymorphisms Are Associated with Human Allergy. PLoS ONE.

[57] Dong J., Liao W., Peh H.Y., Chan T.K., Tan W.S.D., Li L., Yong A., Wong W.S.F. (2017). Ribosomal protein S3 gene silencing protects against experimental allergic asthma. Br. J. Pharmacol..

[58] Kidd C.D.A., Thompson P.J., Barrett L., Baltic S. (2015). Histone Modifications and Asthma. The Interface of the Epigenetic and Genetic Landscapes. Am. J. Respir Cell Mol. Biol..

[59] Wan F., Lenardo M.J. (2010). The nuclear signaling of NF-κB: Current knowledge, new insights, and future perspectives. Cell Res..

[60] Zhou X., Kinlough C.L., Hughey R.P., Jin M., Inoue H., Etling E., Modena B.D., Kaminski N., Bleecker E.R., Meyers D.A. (2019). Sialylation of MUC4β N-glycans by ST6GAL1 orchestrates human airway epithelial cell differentiation associated with type-2 inflammation. JCI Insight.

[61] Lachowicz-Scroggins M.E., Yuan S., Kerr S.C., Dunican E.M., Yu M., Carrington S.D., Fahy J.V. (2016). Abnormalities in MUC5AC and MUC5B Protein in Airway Mucus in Asthma. Am. J. Respir Crit. Care Med..

[62] Balbisi M., Sugár S., Schlosser G., Szeitz B., Fillinger J., Moldvay J., Drahos L., Szász A.M., Tóth G. (2023). Inter- and intratumoral proteomics and glycosaminoglycan characterization of ALK rearranged lung adenocarcinoma tissues: A pilot study. Sci. Rep..

[63] Tyanova S., Temu T., Cox J. (2016). The MaxQuant computational platform for mass spectrometry-based shotgun proteomics. Nat. Protoc..

[64] Cox J., Neuhauser N., Michalski A., Scheltema R.A., Olsen J.V., Mann M. (2011). Andromeda: A peptide search engine integrated into the MaxQuant environment. J. Proteome Res..

[65] Jacob D., Deborde C., Moing A. (2020). BioStatFlow -Statistical Analysis Workflow for ‘Omics’ Data. arXiv.

