# Supplementary material for: Interleukin-13 Treatment of Living Lung Tissue Model Alters the Metabolome and Proteome—A Nano-DESI MS Metabolomics and Shotgun Proteomics Study
Source: Int J Mol Sci. 2024 May 5;25(9):5034. doi: 10.3390/ijms25095034 (PMC11084154; doi:10.3390/ijms25095034)
Supplement: Supplementary file 1 [file ijms-25-05034-s001.zip › ijms-2928257-supplementary.pdf]

Supplementary information

To the article titled:

Interleukin-13 Treatment of Living Lung Tissue Model Alters the Metabolome and Proteome – a nano-DESI MS Metabolomics and Shotgun Proteomics Study

Gábor Tóth[1], Anastasia Golubova[1], Alexander Falk[1], Sara Bergström Lind[1], Mark Nicholas[2], Ingela Lanekoff[1*]

1) Department of Chemistry-BMC, Uppsala University, Sweden

2) AstraZeneca R&D, Mölndal, Sweden

[*]**Correspondence:** Ingela.Lanekoff@kemi.uu.se

Table of contents

1. Supplementary figures

Figure S1 Apical histamine signal as a function of basal histamine concentration.

Figure S2 Intensity of histamine and methyl-histamine during drug exposure.

Figure S3 Volcano plot of all quantified proteins with indication of highly differentially expressed proteins.

2. Supplementary tables

Table S1 List of annotated endogenous metabolites and intensity ratios between the two sample groups at the given time points.

Table S2 List of significantly upregulated proteins in the Treated sample group compared to Control.

Table S3 List of significantly downregulated proteins in the Treated sample group compared to Control.

Table S4 Uniquely detected proteins in Treated and Control sample groups.

1. Supplementary figures

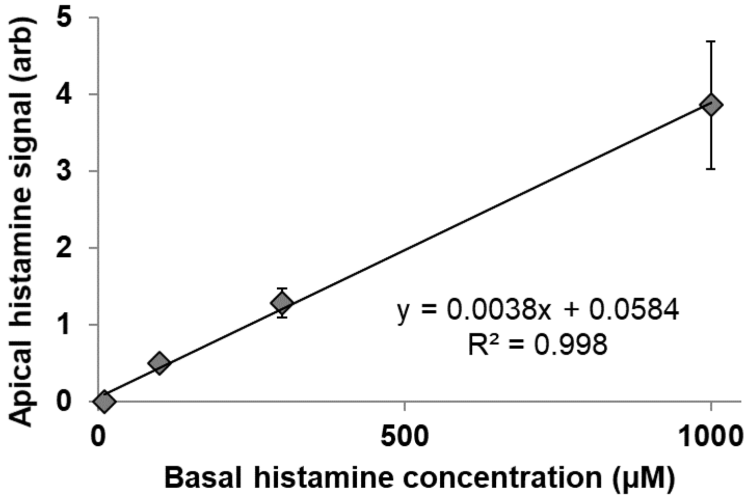

Figure S1 Apical histamine signal as a function of basal histamine concentration. Since a linear function was observed, the transport can be assumed concentration-independent in the investigated concentration range.

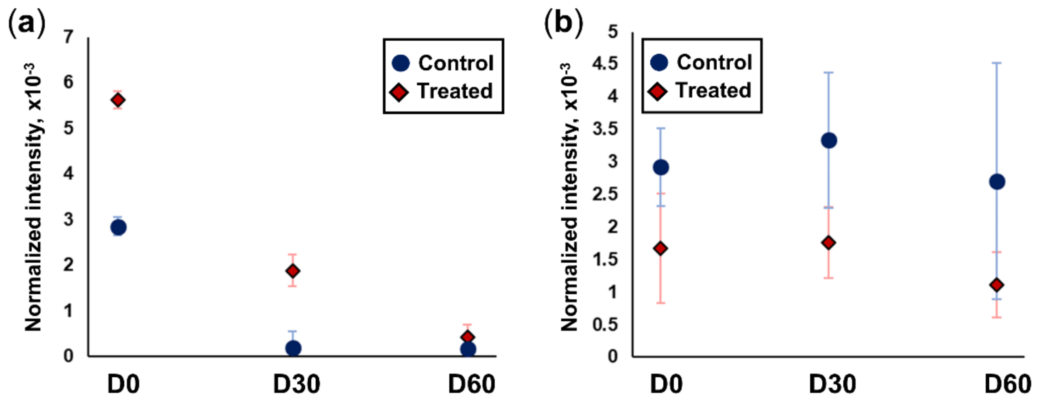

Figure S2 Intensity of histamine (**a**) and methylhistamine (**b**) during drug exposure. Intensities normalized to the TIC are shown, and error bars represent standard deviation.

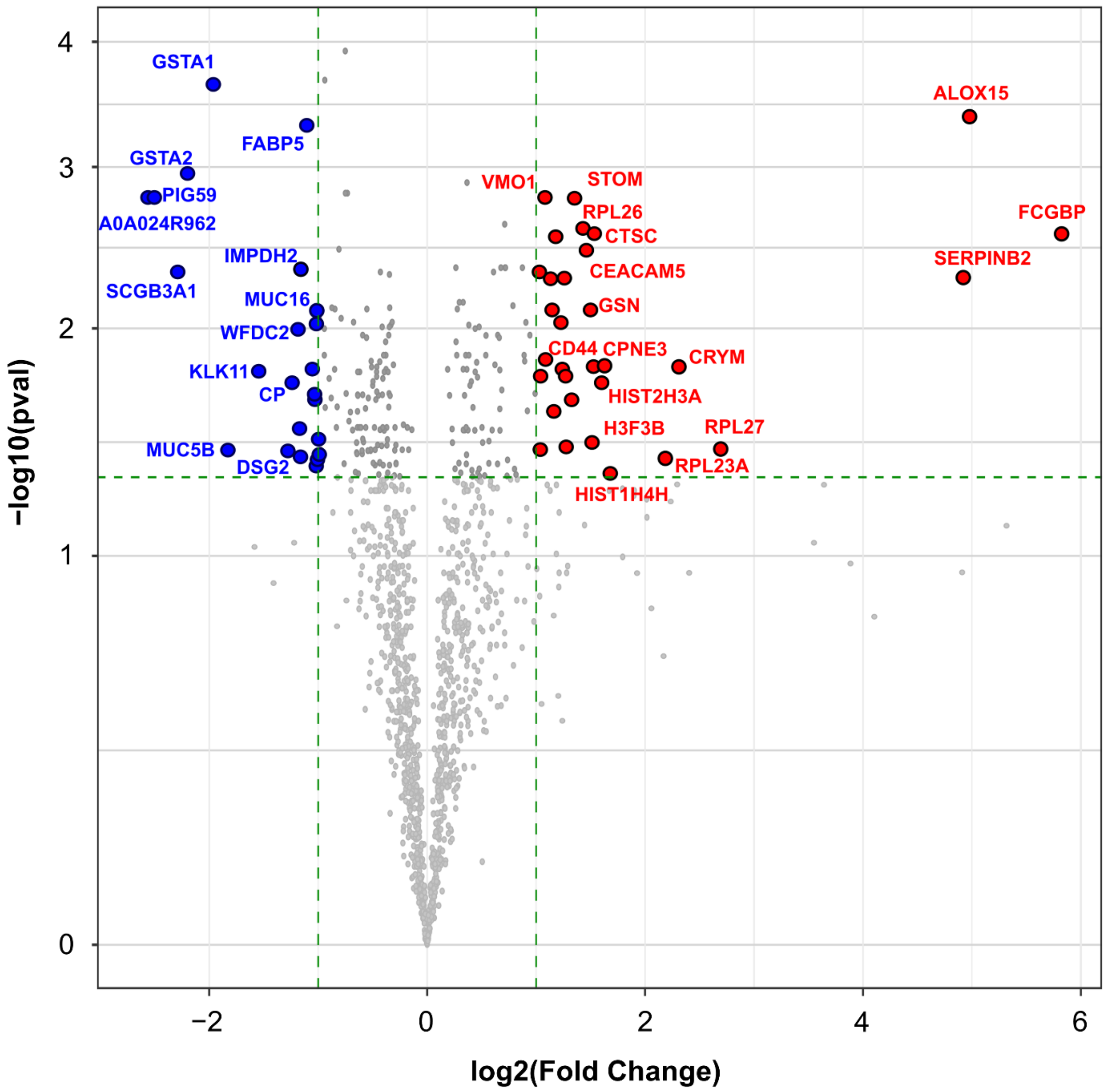

Figure S3 Volcano plot of all quantified proteins with indication of highly differentially expressed proteins. The vertical dashed lines denote a 2-fold change while the horizontal dashed line denotes p = 0.05 significance threshold. Blue – significantly lower in the Treated sample group, Red – significantly higher in the Treated sample group.

2. Supplementary tables

Table S1 List of annotated endogenous metabolites and intensity ratios between the two sample groups (Treated *over* Control, I_T/I_C, [-]) at the given time points. (uC: uniquely detected in the Control sample group, uT: uniquely detected in the Treated sample group).

**Feautre**	**H0**	**H30**	**H60**	**D0**	**D30**	**D60**
[Phosphocholine]$^+$ or [Dehydroxycarnitine+K]$^+$	0.36	1.16	0.23	0.50	0.41	0.40
[Creatinine+Na]$^+$	0.46	0.45	0.45	0.43	0.31	0.56
[Creatine+H]$^+$	*uC*	0.52	0.24	*uC*	0.32	0.48
[Creatine+Na]$^+$	*uT*	0.43	0.41	0.44	0.30	0.47
[Creatine+K]$^+$	*uT*	0.56	0.37	0.52	0.33	0.62
[Gln+H]$^+$	0.77	1.52	0.50	0.62	0.63	1.01
[Gln+K]$^+$	0.79	1.92	0.70	0.86	0.86	1.22
[Gln+Na]$^+$	1.15	1.20	0.95	0.71	0.83	1.00
[Taurine+K]$^+$	0.92	1.96	0.72	0.97	0.79	1.21
[Leu/Ile+Na]$^+$	0.93	0.93	2.43	1.37	1.67	1.61
[Choline]$^+$	1.28	0.92	1.08	1.38	1.44	1.07
[Trp+Na]$^+$	1.34	1.55	1.19	1.04	1.18	1.11
[Taurine+Na]$^+$	1.49	1.31	0.99	0.80	0.66	0.86
[Lys+H]$^+$	1.93	1.14	1.10	1.11	1.23	1.22
[Pro+Na]$^+$	3.14	1.02	1.16	0.88	0.99	0.73
[Hypoxanthine+K]$^+$	5.16	1.63	1.23	2.84	1.36	*uT*
[Hypoxanthine+Na]$^+$	*uT*	1.55	*uT*	3.99	1.45	1.57
[Tyr+Na]$^+$	*uT*	0.71	1.21	1.03	1.39	1.17
[His+H]$^+$	1.26	1.75	0.86	0.85	0.86	1.37
[His+Na]$^+$	*uT*	0.90	1.56	0.99	1.37	1.25
[Adenine+H]$^+$	*uT*	1.00	0.94	1.53	1.12	0.80
[Val+Na]$^+$ or [Betaine+Na]$^+$	*uT*	1.22	2.48	1.41	1.65	*uT*
[Glucose+K]$^+$	1.12	1.20	1.12	1.42	1.09	1.74
[Glucose+Na]$^+$	*uT*	1.23	1.44	1.24	1.26	1.25
[Ser+Na]$^+$	*uT*	1.31	1.22	1.05	0.91	1.37
[Oxoproline+H]$^+$	*uT*	1.39	0.56	*uC*	0.82	0.96
[Oxoproline+Na]$^+$	1.19	1.21	0.98	0.72	0.93	0.97
[Oxoproline+K]$^+$	*uT*	2.09	0.77	1.15	1.05	1.22
[GPC+H]$^+$	*uT*	2.55	0.58	2.54	1.07	1.35
[GPC+K]$^+$	1.26	3.08	1.35	1.87	1.25	1.84
[GPC+Na]$^+$	1.74	2.07	1.49	1.07	1.27	1.68
[Pro+K]$^+$	*uT*	*uT*	1.08	*uC*	0.96	1.08
[Thr+Na]$^+$	*uT*	*uT*	1.62	1.27	1.18	1.64
[Met+Na]$^+$	*uT*	*uT*	*uT*	1.21	1.16	*uT*
[Phe+Na]$^+$	1.80	1.18	1.72	1.26	1.57	1.41
[Phe+K]$^+$	*uT*	*uT*	*uT*	*uC*	1.11	*uT*
[Arg+H]$^+$	2.95	2.11	2.25	1.38	1.93	2.21
[Cit+K]$^+$	*uT*	*uT*	*uT*	*uT*	*uT*	*uT*
[Cit+Na]$^+$	*uT*	*uT*	*uT*	*uT*	*uT*	*uT*

Table S2 List of significantly upregulated proteins (FC>2, BH-corrected p-value < 0.05) in the Treated sample group compared to Control.

Protein name	Gene name	Fold Change (Treated vs Control)	p-value
IgGFc binding protein	FCGBP	54.7	0.0026
Arachidonate 15 lipoxygenase	ALOX15	30.5	0.0004
Plasminogen activator inhibitor 2	SERPINB2	29.3	0.0049
60S ribosomal protein L27	RPL27	6.3	0.0363
Ketimine reductase, µ-Crystallin	CRYM	4.8	0.0150
60S ribosomal protein L23a	RPL23A	4.4	0.0399
Histone H4	HIST1H4H	3.2	0.0463
Copine 3	CPNE3	3.0	0.0148
Histone H3 2	HIST2H3A	3.0	0.0180
CD44 antigen	CD44	2.8	0.0150
Dipeptidyl peptidase 1	CTSC	2.8	0.0026
Histone H3 1	H3F3B	2.8	0.0341
Gelsolin	GSN	2.7	0.0075
Carcinoembryonic antigen related cell adhesion molecule 5	CEACAM5	2.7	0.0034
60S ribosomal protein L26	RPL26	2.7	0.0024
Erythrocyte band 7 integral membrane protein	STOM	2.5	0.0015
Histone H3 1	HIST1H3A	2.4	0.0218
40S ribosomal protein S13	RPS13	2.4	0.0357
Small nuclear ribonucleoprotein Sm D1	SNRPD1	2.3	0.0167
60S ribosomal protein L32	RPL32	2.3	0.0049
60S ribosomal protein L14	RPL14	2.3	0.0154
Tetraspanin	CD9	2.3	0.0088
Annexin 3	HEL.S.274	2.2	0.0028
40S ribosomal protein S24	RPS24	2.2	0.0247
Plastin 1	PLS1	2.1	0.0075
Galectin	hCG_22119	2.1	0.0050
Beta galactoside alpha 2,6 sialyltransferase 1	ST6GAL1	2.1	0.0138
Vitelline membrane outer layer protein 1 homolog	VMO1	2.0	0.0015
ATP dependent 6 phosphofructokinase platelet type	PFKP	2.0	0.0167
40S ribosomal protein S25	RPS25	2.0	0.0367
Complement factor B	CFB	2.0	0.0045

Table S3 List of significantly downregulated proteins (FC<0.5, BH-corrected p-value < 0.05) in the Treated sample group compared to Control. In the third column, the reciprocal of the FC value is displayed for each protein.

Protein name	Gene name	Fold Change (Control vs Treated)	p-value
Complement factor H	A0A024R962	6.10	0.0015
Glutamine synthetase	PIG59	5.76	0.0015
Secretoglobin family 3A member 1	SCGB3A1	5.05	0.0045
Glutathione S transferase		4.66	0.0010
Glutathione S transferase A1	GSTA1	3.96	0.0002
Mucin 5B	MUC5B	3.67	0.0368
Kallikrein 11	KLK11	3.02	0.0158
Desmoglein 2	DSG2	2.51	0.0372
Ceruloplasmin	CP	2.44	0.0180
WAP four disulfide core domain protein 2	WFDC2	2.35	0.0096
Transmembrane channel like protein 1	TMC5	2.33	0.0296
Prolactin inducible protein	PIP	2.31	0.0394
Inosine 5 monophosphate dehydrogenase 2	IMPDH2	2.31	0.0045
Fatty acid binding protein epidermal	FABP5	2.18	0.0005
Prominin 1	CD133	2.15	0.0154
Calcium and integrin binding protein 1	CIB1	2.11	0.0205
Latexin	LXN	2.08	0.0405
Sulfhydryl oxidase 1	QSOX1	2.05	0.0090
Retinoic acid receptor responder protein 1	RARRES1	2.05	0.0385
Mucin 16	MUC16	2.05	0.0078
EH domain containing protein 4	EHD4	2.02	0.0431
Bifunctional ATP dependent dihydroxyacetone kinase	DAK	2.00	0.0218

Table S4 Uniquely detected proteins in Treated and Control sample groups.

**TREATED**		
**Protein names**	**Number of unique peptides**	**Average LFQ value**
Nitric oxide synthase, inducible	23	1.99E+08
Serpin B10	20	6.51E+08
UDP-N-acetylglucosamine pyrophosphorylase	16	2.24E+08
Histone H1.0, N-terminally processed	15	4.81E+08
Histone H1.2	14	2.67E+08
Protein-glutamine gamma-glutamyltransferase 2	14	1.34E+08
Dehydrogenase/reductase SDR family member 7	13	1.31E+08
Fetuin-B	13	1.11E+08
Cystatin-SN	12	6.32E+09
60S ribosomal protein L19	11	2.36E+08
Solute carrier family 12 member 2	11	8.94E+07
Alkaline phosphatase	10	1.83E+08
60S ribosomal protein, L36a-like	8	1.17E+08
Multidrug resistance-associated protein 1	8	3.96E+07
Carbonic anhydrase 2	7	6.99E+07
DCC-interacting protein 13-beta	6	1.12E+08
15-hydroxyprostaglandin dehydrogenase [NAD(+)]	6	9.64E+07
Lysyl oxidase homolog 4	6	8.81E+07
Carbohydrate sulfotransferase 4	5	5.66E+07
Dimethylaniline monooxygenase, N-oxide-forming	4	4.88E+07
Peroxisomal membrane protein 11B	4	1.94E+07

**CONTROL**		
**Protein names**	**Number of unique peptides**	**Average LFQ value**
Outer dense fiber protein 3B	9	6.27E+07
Disintegrin and metalloproteinase domain-containing protein 10	8	6.76E+07
Plakophilin-2	8	3.87E+07
SAFB-like transcription modulator	7	3.50E+07
